# Carbapenemase-producing uropathogens in real life: epidemiology and treatment at a County Emergency Hospital from Eastern Romania

**DOI:** 10.25122/jml-2023-0139

**Published:** 2023-05

**Authors:** Aurel Rusu, Catalin Tiliscan, Aida-Isabela Adamescu, Oana-Alexandra Ganea, Victoria Arama, Stefan Sorin Arama, Stefan Alexandru Rascu, Viorel Jinga

**Affiliations:** 1. Carol Davila University of Medicine and Pharmacy, Bucharest, Romania; 2. Department of Urology, Vaslui Emergency County Hospital, Vaslui, Romania

**Keywords:** carbapenemase, epidemiology, nonacademic hospital, treatment, uropathogens, AMR: Antimicrobial resistance, CA-UTI: Catheter-associated urinary tract infection, CRE: Carbapenem-resistant Enterobacterales, CREC: Carbapenem-resistant E. coli, CRKP: Carbapenem-resistant Klebsiella pneumoniae, DTR: Difficult-to-treat, ICU: Intensive care unit, ID: Infectious disease, MDR: Multi-drug resistant, SIRS: Systemic inflammatory response syndrome, US: United States, UTI: Urinary tract infections, XDR: Extended drug-resistant

## Abstract

Urinary tract infections are a public health problem exacerbated by the rising concern of antibiotic resistance. Carbapenem-resistant Enterobacterales (CRE), mostly isolated from urine samples, represent an immediate public health threat, often associated with healthcare settings. This study investigated 27 cases of carbapenemase-producing organisms (CPO) detected in urinalysis over one year. There was a significant association between the presence of chronic indwelling urinary catheters and the temporary use of urinary catheters, with both groups accounting for 66.7% of all cases. We identified two modes of transmission for extended drug-resistant microorganisms: inter-hospital spread, covering wide geographical distances (involving four healthcare units across two other counties), and intra-hospital transmission (12 departments within our institution). Medium-size hospitals should thoroughly investigate their specific carbapenemase-producing strains. Their laboratories must be well-supplied to handle this situation and perform the necessary testing accurately. Treatment options should be available based on presumed susceptibility and antimicrobial susceptibility testing, with a range of antibiotics available, including novel agents such as Ceftazidime-avibactam, as well as established options like Aminoglycosides and Colistin. Adherence to rigorous catheter handling protocols, as emphasized by national and international guidelines, is essential and should be implemented consistently across all hospital departments.

## INTRODUCTION

Urinary tract infections (UTIs), which are estimated to afflict up to 150 million people per year globally [1], are a significant public health concern. They are the second most prevalent infectious diseases in medical practice. The wide use of antibiotics adds significantly to this problem, increasing the risk of bacterial antimicrobial resistance emergence. The development of antibiotic resistance is a natural process that occurs when bacteria adapt to the selective pressure exerted by antibiotics. Misuse and overuse of antibiotics can accelerate this process, leading to the emergence of antibiotic-resistant strains of bacteria that are more difficult to treat. Considering that the discovery of new antibiotics has slowed down significantly, and antibiotic use has increased, antibiotic resistance has become an increasingly serious issue in recent years [2].

Bacterial antimicrobial resistance (AMR), which occurs when bacteria develop mechanisms to counteract the effects of antibiotics, making them less effective or completely ineffective, has emerged as one of the leading public health threats of the 21^st^ century [3]. AMR imposes a significant clinical and public health burden, and as this burden is expected to grow over time, immediate action is needed. The AMR Review [2] acknowledges that the reported numbers are “broad brush estimates” and emphasizes the need for “more detailed and robust work” to be conducted by academic researchers. This highlights the current lack of comprehensive data on AMR, underscoring the importance of improving infection surveillance [4]. This lack of data is particularly prevalent in nonacademic medical entities, where limited research is conducted despite their significant impact on society's healthcare.

The current data in the European Union (EU) highlights the emergence of resistance among Gram-negative bacteria as a pressing concern that will likely present significant challenges in the years ahead [5]. Carbapenem-resistant Enterobacterales (CRE) represent an immediate public health threat that requires urgent and aggressive action due to their resistance to most antibiotics and poor clinical outcomes. While carbapenem-resistant *Klebsiella pneumoniae* (CRKP) is more commonly reported, the emergence of carbapenem-resistant *E. coli* (CREC) is increasing globally. Outbreaks of CRE have been reported in the United States (US), and it is well-established that most CRE infections in both the US and Europe are associated with healthcare settings, with patients in long-term care facilities being at high risk [6].

Most studies have reported that CRE is predominantly isolated from urine samples, with patients in extended care facilities and those with chronic indwelling urinary catheters at the highest risk [7]. The high prevalence of these illnesses is a matter of significant concern. According to estimates provided by Temkin, the number of worldwide infections caused by antibiotic-resistant *E. coli* and *K. pneumoniae* in 2014 was significant. Specifically, approximately 50.1 million serious infections were resistant to third-generation cephalosporins, and 3.1 million serious infections were resistant to carbapenems, as estimated using their additive model [8]. In Romania, studies have shown a significant correlation between novel resistance patterns such as difficult-to-treat (DTR) or carbapenem-resistant Enterobacterales (CRE) and poor patient outcomes in UTIs. Furthermore, it has been observed that most CRE uropathogens in Romania are carbapenemase-producing [9]. Therefore, understanding these resistance patterns becomes crucial in guiding more appropriate and effective approaches.

The study aimed to evaluate the antibiotic resistance patterns, source of contamination, and the clinical impact of urinary tract infections (UTIs) caused by carbapenemase-producing uropathogens in a non-academic clinical facility in Romania.

## MATERIAL AND METHODS

### Study design and setting

A retrospective study was conducted at the Vaslui County Emergency Hospital in Romania from October 1^st^, 2021, to September 30^th^, 2022. The study included patients with urinary tract infections (UTIs) admitted to or appointed for treatment at the hospital. Hospitalized and non-hospitalized patients were included, and clean catch midstream urine samples were collected as standard practice.

### Sample collection and processing

The urine samples were collected in wide-mouth sterile containers and processed immediately using the usual procedure. The specimen was considered positive if a single organism was cultured at >100.000 CFU/ml concentration. The isolated colonies were then tested for antimicrobial susceptibility using the disc diffusion technique on agar after a standard inoculum was swabbed on its surface using the ready-made antibiotics-supplied dispenser discs [10]. The central laboratory of the hospital performed all urine cultures and susceptibility tests. The selection criterion for this study was the presence of bacteriuria with carbapenemase-producing bacteria, specifically the formation of more than 100,000 colonies of carbapenem-resistant Enterobacterales (CRE). Thus, all cases with positive urine cultures containing carbapenemase-producing uropathogens were included in the analysis [11].

### Data collection

Socio-demographic data including sex, age, place of residence, and clinical and epidemiological details such as department, Intensive Care Unit (ICU) admission, diagnosis, the presence of systemic inflammatory response syndrome (SIRS), isolated bacteria, antibiogram, treatment, urinary catheter placement, control-culture, death occurrence, previous hospitalizations, and the probable place of contamination were obtained for each patient. The variables associated with each case were gathered from patient charts, the informatic system of the hospital, discussions with the attending physicians, and attempts to contact the patients or their caregivers (this last approach had little impact as most contacted persons could not respond adequately to the questions).

### Data analysis

Data were analyzed using Microsoft Excel software and the Statistical Package for the Social Sciences (SPSS) version 22 (IBM, Armonk, NY, United States of America). Descriptive statistics were calculated, and percentages were derived for certain variables.

### Results

During the one-year study conducted at Vaslui County Emergency Hospital, 27 patients with CPO were identified and included in the study. The mean age of the cohort was 65.4±18.1 years, with a range of less than one year and a maximum of 85 years old. The majority of patients were male (77.8%), with only 22.2% females (6 females and 21 males). More than half of the patients (59.3%) were rural residents, and 40.7% had an urban residence.

#### Microorganisms, antibiotic susceptibility, and treatment

Among the isolated microorganisms, the most frequently identified were *Pseudomonas aeruginosa* (8 cases – 29.6%), *Klebsiella pneumoniae* (8 cases – 29.6%), *Klebsiella terrigena* (5 cases -18.5%), *Klebsiella oxytoca* (2 cases – 7.4%), *Escherichia coli* (3 cases – 11.1%), and *Acinetobacter baumanii* (one – 3.7%). Klebsiella spp. accounted for over half of all cases (55.5%).

In accordance with the inclusion criteria, 96.3% of the tested bacterial strains were carbapenem-resistant, and 3.7% were carbapenem-intermediate.

Most patients (88.9%) were never tested for colistin susceptibility, two patients were positive for colistin-resistant microorganisms, and only one (3.7%) had a colistin-susceptible strain. Most of the tested microorganisms (88.9%) were ceftazidime-avibactam susceptible, while two patients (7.4%) had ceftazidime-avibactam-resistant microorganisms, and one person was never tested (Table 1). For ceftazidime alone, 85.2% of the isolates were resistant, 7.4% susceptible, and 7.4% untested. Fosfomycin testing was conducted for three bacterial strains (11.1%), all susceptible.

**Table 1.** Antibiotic sensitivity testing for carbapenemase-producing organisms; Aminoglycosides -AG, Gentamycin-G, Amikacin-A, Fosfomycin-F

	Colistin	Ceftazidime/Avibactam	Aminoglycosides + Fosfomycin	Meropenem	Cefepime	Ceftazidime	Piperacillin - Tazobactam	Levofloxacin	Cefiderocol
			G	A	F						
Susceptible	3.7%	88.9%	44.4%	59.3%	11.1%		3.7%	7.4%	11.1%	3.7%	18.5%
Intermediate						3.7%			3.7%		
Resistant	7.4%	7.4%	40.7%	40.7%	0	96.3%	25.9%	85.2%	77.8%	96.3%	
Not tested	88.9%	3.7%	14.8%		88.9%		70.4%	7.4%	7.4%		81.5%

All strains were tested for amikacin, 16 were sensitive, and 11 were resistant (59.3% sensitivity and 40.7% resistance, respectively). Only 23 out of 27 were tested for gentamycin, and 12 were sensitive, while 11 were resistant (44.4% sensitivity and 40.7% resistance, respectively). Over half of the patients (70.4%) did not undergo cefepime susceptibility testing; one strain (3.7%) was susceptible, and 25.9% were resistant. Piperacillin/tazobactam showed 77.8% resistance, 11.1% sensitivity, and 3.7% intermediate resistance, with 7.4% of isolates remaining untested. Ticarcillin/clavulanate testing was not performed for most cases (88.9%), with 7.4% of strains found to be resistant and 3.7% susceptible.

All samples were tested for levofloxacin, and 96.3% of the bacterial strains were resistant, and one case was susceptible (3.7%). Five bacterial strains underwent cefiderocol susceptibility testing in the microbiology laboratory of the hospital, even though this antibiotic was not yet available by the time this study concluded. All 5 strains were cefiderocol-susceptible.

Treatment options were partially guided by the antibiogram findings. The therapy options are listed in decreasing order of frequency. Colistin was the most commonly used antibiotic, administered to 25.9% of patients (7 out of 27 cases). Although colistin was not tested enough, it was frequently used because of the presumed susceptibility. Amikacin was the second most frequently used (22.2%), followed by gentamycin (14.8%) and ceftazidime-avibactam (11.1%). The remaining cases were treated with piperacillin-tazobactam, levofloxacin, or tigecycline. Four patients were lost to follow-up.

#### Clinical evolution and outcome

More than half of the patients (55.6%) met the criteria for systemic inflammatory response syndrome (SIRS), and 22.2% were admitted to the ICU due to the severity of their condition. One death was reported (3.7%), while the rest of the patients had a favorable outcome and were discharged.

Approximately one-third (29.6%) of enrolled subjects had chronic indwelling urinary catheters (8 patients). One case was discharged from another hospital with bladder drainage and a recommendation for eventual removal but developed pyuria. Two cases involved upper urinary tract drainages: one internal JJ stent and one ureteral stent on a cutaneous ureterostomy. A urinary catheter was placed for 22.2% of the patients during the healthcare unit admission. A quarter of the patients (25.9%) did not undergo urinary catheterization.

The clinical diagnosis for most patients was urinary tract infection (UTI), with 29.6% reported as catheter-associated UTI (CA-UTI). One patient had Fournier gangrene (3.7%), with a good clinical outcome. Control urine culture was collected in 11 out of 27 cases (40.7%), and all were negative.

#### Epidemiology

The designated source of infection for 51.9% of enrolled patients was the Vaslui County Emergency Hospital. The remaining cases were associated with four other healthcare units in two counties (Iasi and Bucharest). The patients from these two cities came from three departments: urology, neurology, and oncology. Furthermore, seven of these cases were discharged from urology services, suggesting that 25.9% of all cases (from the three hospitals outside Vaslui County) were contaminated in urology departments.

Upon examining the distribution of carbapenemase-producing strains within the hospital, 11 departments were identified, excluding the ICU. The department with the highest number of cases was Infectious Disease, accounting for 29.6% of the total cases. The Emergency Care Unit and the hospital ambulatory each had 11.1% of the cases. Other departments included Pneumology (7.4%), General Surgery (7.4%), Urology (7.4%), Neurology (7.4%), Internal Medicine (7.4%), Gastroenterology (3.7%), Pediatrics (3.7%), and Diabetes (3.7%).

### DISCUSSION

The literature presents conflicting findings regarding the role of sex as a risk factor for drug-resistant UTIs. While some studies have not found significant differences in antibiotic susceptibility among Enterobacterales isolated from males and females in common UTIs [12], our study observed a higher proportion of male patients, which aligns with findings from other authors [13,14]. This is probably due to coexistent bladder obstruction pathology, such as prostate and urethral diseases, and the increased risk of urinary retention and consecutive catheter presence. While there may not be gender differences in larger cohorts of common UTIs, our subgroup of XDR UTIs demonstrates a significant male predominance.

The mean age of the cohort exceeded 65 years, in accordance with other authors indicating that older age increases the likelihood of acquiring an antibiotic-resistant urinary tract infection [12-14].

Regarding antibiotic sensitivity testing in the study cohort, we report that most patients were never tested for colistin susceptibility due to a shortage of laboratory supplies following the COVID-19 pandemic in a hospital located in one of the most underdeveloped regions of Romania. Similarly, most of the isolates were not tested for Fosfomycin sensitivity. While the limited number of tested isolates showed 100% sensitivity, the small sample size limits the statistical significance of this finding.

However, we emphasize that Fosfomycin is accessible almost exclusively in oral form in Romania, and intravenous Fosfomycin might represent a valuable alternative for CRE UTIs. In addition, cefiderocol might be an important treatment option in the near future in most cases [15]. Ceftazidime-avibactam should be used judiciously as a last resort, according to antibiotic stewardship, considering its significance in the current and future treatment landscape [16].

Colistin, gentamicin, and amikacin play crucial roles in the treatment and clinical outcome of CRE UTIs [17,18]. The lack of colistin testing presents a significant limitation in the present study and is a common issue in some hospitals. It is important to address and improve this limitation. However, Colistin had a major role in the treatment of this cohort and contributed significantly to the good cure rate. Approximately half of the patients had CRE strains sensitive to aminoglycosides, highlighting the continued importance of these antibiotics as treatment options. Among the strains tested, approximately 50% showed sensitivity to gentamicin. However, ceftazidime, piperacillin-tazobactam, and fluoroquinolones, which were extensively used in the past, are no longer suitable options due to high levels of resistance exceeding 80%.

Our investigation into the mechanism of infection revealed the involvement of urinary catheters. Of the 27 cases examined, 18 patients had either a current or previous catheter, which could be attributed to factors such as urological surgical interventions during previous hospital admissions. These percentages differ from the ones described above (where we just roughly considered the catheter presence during admission in Vaslui Hospital) because, in some instances, the catheter was inserted during other hospital admissions and removed there, or it was inserted during the same admission for caring reasons despite the presence of a known urinary tract infection. After thoroughly putting events in order, it was concluded that approximately 66.7% of all cases were likely caused by catheter insertion/presence. The other 9 patients, representing 33.3% of all cases, did not have a history of catheterization that could have led to contamination, and the precise mechanism by which bacteria penetrated their urinary tracts remains unknown. Consequently, we can emphasize the strong correlation between urinary tract catheterization and healthcare-associated infections, as the majority of cases in our study can be attributed to this factor. Furthermore, besides bladder draining, 7.4% of the cases involved instrumentation of the upper urinary tract, consistent with findings from other studies that have identified it as a potential risk factor [19].

The limited collection of control urine cultures in our study can be attributed to various factors. Harvesting a control urine culture is not yet a well-established procedure, and some patients may be called back for ambulatory evaluation where urine culture is collected. Physicians' reluctance to discuss the importance of follow-up urine cultures may be influenced by their desire to expedite processes in the face of hospital-acquired infections and anxiety triggered by MDR/XDR bacteria in daily practice [20].

Among the patients included in the study, one death was recorded, representing a mortality rate of 3.7%. It is important that this event occurred during the New Year's Eve holidays when attending physicians and ICU staff were frequently rotated. The lack of continuity in care and understaffing in the laboratory may have impacted the quality and timeliness of actions and results, potentially affecting the optimal management of the case. Furthermore, the patient's critical condition upon admission and direct transfer to the ICU also contribute to the complexities surrounding this particular case. Although one death cannot provide definitive conclusions, most patients survived, suggesting a favorable prognosis for this disease setting. The rest of the patients had a favorable outcome and were discharged.

Our findings indicate a significant circulation of carbapenem-resistant bacteria between healthcare units located at considerable distances. In this study, the source of infection was identified in hospitals from Vaslui, Iasi, and Bucharest, spanning 80 and 350 kilometers. Even medium size hospitals harbor dangerous XDR bacteria, including carbapenemase-producing strains. In two cases, we could not identify any contact with any healthcare unit before infection. Immediate actions, more effective strategies, and improved surveillance are needed to confine and prevent the further spread of multidrug-resistant Gram-negative pathogens in healthcare institutions, particularly concerning the inter-hospital aspect [21].

Examining the distribution of these strains within the hospital revealed that the risk of encountering or being contaminated by these dangerous microbes extends across various departments, ranging from Infectious Diseases to Pediatrics. Each department must be prepared to manage such situations, emphasizing the importance of rigorous catheter handling and adherence to guidelines, such as the European and Asian guidelines for managing and preventing catheter-associated urinary tract infections [22].

Therefore, judicious management of antibiotic treatment is essential in limiting the spread of resistance. One general guideline is not satisfactory, as adapting antibiotic treatment to local resistance patterns provides better results and is generally recommended [23]. Improved antibiotic testing, better infection control, and antibiotic stewardship programs should be an integral part of the management of these severe infections.

The study has several limitations. Some patients had lengthy and complex medical histories spanning many years, and complete personal medical histories were unavailable for some non-hospitalized patients. There may have been hesitancy among the medical staff to discuss this subject. Laboratory limitations, particularly the insufficient testing of colistin and Fosfomycin susceptibilities. Furthermore, nitrofurantoin was not tested in the antibiogram, and it should have been, as it is presumed that many bacterial strains are still sensitive to this drug. Moreover, there are limitations regarding the treatment because neither the IV form of Fosfomycin is available for use in the institution nor the oral form.

## CONCLUSION

The current study revealed two modes of CRE transmission: inter-hospital and intra-hospital pathways, both requiring enhanced control measures. A significant number of patients with UTIs caused by CRE were identified, mostly men, with a mean age of over 65 years. While most strains were susceptible to ceftazidime-avibactam and approximately half to aminoglycosides, almost all patients achieved clinical cure after treatment with colistin, aminoglycosides, or ceftazidime-avibactam. The urinary catheter was involved in two-thirds of all patients, so rigorous catheter handling is needed, and the right procedures should be applied as strongly stated by many guideline recommendations.
